# The CDK4/6 inhibitor abemaciclib attenuates cognitive impairment and neuroinflammation via DYRK1A in human tau transgenic mice

**DOI:** 10.1186/s13041-026-01318-x

**Published:** 2026-06-01

**Authors:** Hyun-ju Lee, Hyang-Sook Hoe

**Affiliations:** 1 Department of Neural Development and Disease, Korea Brain Research Institute (KBRI), 61, Cheomdan-ro, Dong-gu, Daegu, 41062 Republic of Korea; 2 AI-based Neurodevelopmental Diseases Digital Therapeutics Group, Korea Brain Research Institute (KBRI), 61, Cheomdan-ro, Daegu, 41062 Republic of Korea; 3 Department of Brain and Cognitive Sciences, Daegu Gyeongbuk Institute of Science & Technology, Daegu, 42988 Republic of Korea

**Keywords:** Abemaciclib, Neuroinflammation, Tau, DYRK1A, Microgliosis, Alzheimer's disease

## Abstract

We recently demonstrated that abemaciclib treatment modulates cognitive function, Alzheimer's disease (AD) pathology, and neuroinflammatory responses in wild-type mice treated with lipopolysaccharide and in 5xFAD mice. In this study, we investigated the influence of abemaciclib treatment on neuroinflammation and cognitive function in 6- or 9-month-old PS19 mice, a P301S mutant tauopathy model. We found that abemaciclib administration suppressed microglial activation in 6-month-old PS19 mice, whereas astrocytic activation was partially attenuated in the entorhinal cortex but not in the hippocampus. In addition, abemaciclib treatment improved short-term and recognition memory and the dendritic spine formation in 6- and 9-month-old PS19 mice. More importantly, abemaciclib administration enhanced short-term and recognition memory in a DYRK1A-dependent manner in 6-month-old PS19 mice. Collectively, our results suggest that abemaciclib treatment alleviates neuroinflammatory responses and cognitive impairment through DYRK1A in 6- or 9-month-old human tau transgenic PS19 mice, highlighting how this multi-kinase-targeting drug could be leveraged for the treatment of neurodegenerative diseases.

**Supplementary Information:**

The online version contains supplementary material available at 10.1186/s13041-026-01318-x.

The predominant form of dementia is Alzheimer’s disease (AD), which manifests chiefly as progressive deficits in learning and memory [1]. Patients with AD develop extracellular senile plaques comprising amyloid beta (Aβ) and intracellular neurofibrillary tangles (NFTs) composed of hyperphosphorylated tau [2]. Aβ/tau pathologies cause cognitive impairment in AD pathoprogression by exacerbating glial activation and the excessive release of proinflammatory mediators [3]. Despite active efforts to develop disease-modifying drugs for AD, there is no AD therapeutic that can successfully regulate the various pathological symptoms of AD [4]. Most AD therapeutics target a single AD pathology, such as Aβ, tau, the cholinergic system, or neuroinflammation, which may explain their limited therapeutic efficacy. Therefore, multitarget drugs could be a successful strategies for treating AD.

Abemaciclib mesylate is an inhibitor of cyclin-dependent kinase 4 (CDK4) and CDK6, and is approved by the FDA for treatment of HR-( +) and HER2-(−) breast cancer [5]. Interestingly, abemaciclib is also a potent inhibitor of dual specificity tyrosine-phosphorylation-regulated kinase 1A (DYRK1A), which is involved in AD-related pathoprogression, including neuroinflammation, cognitive function, and/or Aβ and tau pathology [6]. This dual inhibition of CDK4/6 and DYRK1A implies that abemaciclib might modulate AD pathologies, and we previously demonstrated that abemaciclib treatment improves cognitive function and reduces Aβ/tau pathology and neuroinflammatory responses in 3- to 4-month-old and 6-month-old 5xFAD mice (early and intermediate stages of AD) and 3- to 4-month-old human mutant P301S tau transgenic (PS19) mice (early stage of tauopathy) [7]. In addition, a recent study further reported that abemaciclib treatment attenuates tau pathology through CDK4/6-independent inhibition of tau kinases (e.g., CaMKII and GSK3β) in App^NL−F^/MAPT knock-in mice, an AD model [8]. Here, we extend our previous work by examining the impact of abemaciclib treatment on neuroinflammation and memory function in 6- and 9-month-old PS19 mice.

Six-month-old PS19 mice were intraperitoneally (i.p.) injected with vehicle (10% DMSO) or abemaciclib (30 mg/kg) daily for 17 days. Subsequent immunofluorescence staining with anti-Iba-1 and anti-GFAP antibodies showed that abemaciclib administration significantly reduced Iba-1 fluorescence intensity in the hippocampal CA1 and Iba-1-labeled area in the entorhinal cortex (EC) in 6-month-old PS19 mice (Fig. 1A–C). Furthermore, abemaciclib treatment significantly decreased GFAP fluorescence intensity and GFAP-labeled area in the EC but not the hippocampus of 6-month-old PS19 mice (Fig. 1D–F). These data indicate that abemaciclib treatment partially diminishes microglial/astroglial activation and hypertrophy in 6-month-old PS19 mice. The region-specific effect of abemaciclib on astrocytic activation may be due to regional differences in blood–brain barrier permeability or in the expression and activity of abemaciclib targets (e.g., DYRK1A, CDK4/6, CaMKII, and GSK3β).

**Fig. 1:**
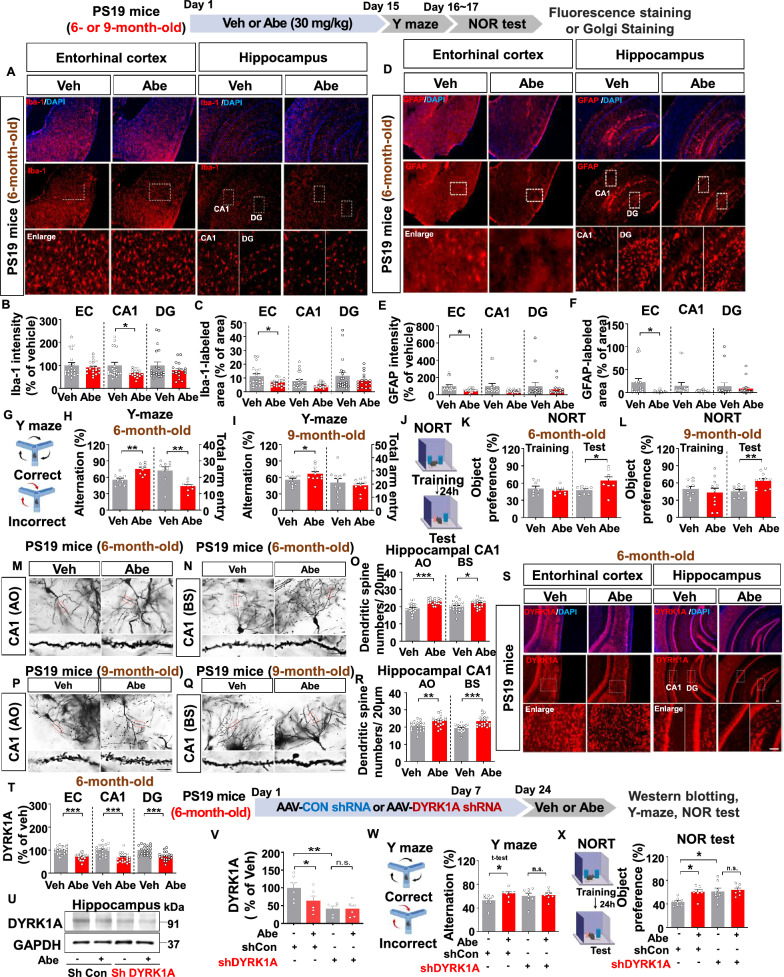
Abemaciclib treatment attenuates tauopathy-mediated microgliosis/astrogliosis and cognitive impairments by modulating DYRK1A in 6- or 9-month-old human tau transgenic PS19 mice. **A–F** 6-month-old PS19 mice were injected with vehicle (10% DMSO, i.p.) or abemaciclib (30 mg/kg, i.p.) daily for 17 days. Immunofluorescence staining was performed with anti-Iba-1 and anti-GFAP antibodies (n = 3–4 brain slices per mouse, 16–20 brain slices from 5 mice/group). Scale bar = 100 µm. **G–L** 6- or 9-month-old PS19 mice were injected with vehicle (10% DMSO, i.p.) or abemaciclib (30 mg/kg, i.p.) daily for 17 days, and Y-maze and novel object recognition (NOR) tests were performed (n = 8–9 mice/group for 6-month-old PS19 mice, n = 10–11 mice/group for 9-month-old PS19 mice). **M–R** After the behavioral tests, Golgi staining of 6- or 9-month-old PS19 mice treated with vehicle (10% DMSO, i.p.) or abemaciclib (30 mg/kg, i.p.) was performed (n = 5 brain slices per mouse, 20 brain slices from 4 mice/group). Scale bar = 10 µm. **S–T** 6-month-old PS19 mice were injected with vehicle (10% DMSO, i.p.) or abemaciclib (30 mg/kg, i.p.) daily for 17 days, and immunofluorescence staining was performed with an anti-DYRK1A antibody (n = 4 brain slices per mouse, 20 brain slices from 5 mice/group). Scale bar = 100 µm. **U** 6- month-old PS19 mice were injected with AAV-CMV-Control shRNA or AAV-CMV-DYRK1A shRNA into the bilateral hippocampal CA1 region. One week after the injection, the mice were injected with vehicle (10% DMSO, i.p.) or abemaciclib (30 mg/kg, i.p.) daily for 17 days. Then, western blotting of hippocampal lysates was performed with an anti-DYRK1A antibody (n = 6 mice/group). **V–W** 6-month-old PS19 mice were injected with AAV-CMV-Control shRNA or AAV-CMV-DYRK1A shRNA into bilateral hippocampal CA1. One week after the injection, the mice were injected with vehicle (10% DMSO, i.p.) or abemaciclib (30 mg/kg, i.p.) daily for 17 days, and Y-maze and NOR tests were conducted (n = 7–8 mice/group). *p < 0.05, **p < 0.01, ***p < 0.001. Statistical analyses were performed using the two-tailed Student’s t-test (**B**,** C**,** E**,** F**,** H**,** I**,** K**,** L**,** O**,** R**,** T** and** W**) and one-way ANOVA (**V** and **X**)

Interestingly, a recent study reported that abemaciclib administration did not alter glial activation in 12-month-old App^NL−F^/MAPT knock-in mice [8]. When considered with previous findings, the current results raise the following question [7, 8]; why does abemaciclib treatment effectively downregulate Aβ/lipopolysaccharide (LPS)-mediated glial activation in 3- to 4-month-old 5xFAD and WT mice but has only minor efficacy in 6-month-old PS19 mice and 12-month-old App^NL−F^/MAPT knock-in mice? It is possible that abemaciclib modulates neuroinflammatory responses more effectively in early-phase tauopathy models because in early disease stages, microglia retain functional plasticity and actively respond to pathological stimuli, allowing their activity to be modulated by pharmacological intervention [9]. By contrast, in advanced AD stages (e.g., 6-month-old PS19 mice or 12-month-old App^NL−F^/MAPT mice), NFT accumulation and associated neuronal injury lead to a shift in glial cell states in which microglia adopt disease-associated phenotypes and induce the formation of neurotoxic reactive astrocytes through inflammatory signaling [10]. These changes sustain gliosis through self-reinforcing microglia–astrocyte signaling, limiting the efficacy of short-term pharmacological intervention [11]. Another possibility is that 17 days of abemaciclib treatment was not sufficient to inhibit gliosis in 6-month-old PS19 mice. A future study will investigate whether treating 6-month-old PS19 mice with abemaciclib for a longer period (e.g., daily for 1 or 2 months) effectively downregulates microglial/astroglial activation.

Chronic neuroinflammation caused by exacerbated glial activation contributes to cognitive impairment, and we recently found that abemaciclib administration rescues memory function in LPS-stimulated WT mice and in Aβ-overexpressing 5xFAD mice [7]. To probe the effects of abemaciclib on cognitive function in human tau transgenic PS19 mice, 6- and 9-month-old PS19 mice were injected with vehicle (10% DMSO) or abemaciclib (30 mg/kg, i.p.) daily for two weeks, and behavior experiments were conducted. The results of Y-maze and novel object recognition (NOR) tests showed that abemaciclib treatment enhanced short-term spatial and recognition memory in 6- and 9-month-old PS19 mice (Fig. 1G–L). In addition, abemaciclib administration significantly increased dendritic spine density in the hippocampal CA1 in 6- and 9-month-old PS19 mice (Fig. 1M–R). These data suggest that abemaciclib treatment improves cognitive function and enhances dendritic spine formation in 6- and 9-month-old PS19 mice.

Although abemaciclib treatment partially reduced gliosis and improved cognitive function, the present data do not establish a direct causal relationship between these effects. Cognitive improvement may arise from multiple mechanisms, including modulation of neuroinflammatory responses, synaptic changes, or parallel signaling pathways. Specifically, while abemaciclib reduced gliosis, the independent contribution of neuroinflammatory modulation to cognitive improvement remains unclear, as glial activity was not selectively manipulated. Therefore, future studies will selectively modulate glial activity (e.g., via CSF1R inhibition or astrocyte/microglia-specific knockdown) to determine whether attenuation of neuroinflammation alone can reproduce the abemaciclib-mediated cognitive improvement. Although dendritic spine density increased in abemaciclib-treated 6- and 9-month-old PS19 mice, the present study did not assess whether synaptic changes are required for the behavioral effects of abemaciclib treatment. To address this, further studies will inhibit NMDA/AMPA receptor-dependent synaptic plasticity (by pharmacological antagonists) or suppress dendritic spine remodeling (by hippocampal neuron-specific knockdown of dendritic spine-regulating proteins) in 6- and 9-month-old PS19 mice, and behavioral tests (e.g., Y-maze and NORT) will be performed to evaluate whether abemaciclib’s cognitive effects are maintained. Lastly, given that abemaciclib targets multiple kinases, including DYRK1A, CDK4/6, CaMKII, and GSK3β, it is possible that its effects on cognition reflect parallel modulation of multiple signaling pathways rather than a single linear mechanism. Therefore, we will employ selective pharmacological inhibition or genetic knockdown of individual kinase targets to determine their contribution to abemaciclib-induced cognitive improvement in 6- and 9-month-old PS19 mice.

DYRK1A is an important molecular target for neuroinflammation and cognitive function, and abemaciclib was recently shown to be a potent DYRK1A inhibitor [6]. To determine whether abemaciclib treatment affects DYRK1A levels in human tau transgenic mice, 6-month-old PS19 mice were injected (i.p.) with vehicle (10% DMSO) or 30 mg/kg abemaciclib daily for 17 days. Subsequent immunofluorescence staining showed that abemaciclib administration significantly decreased DYRK1A expression in the EC and hippocampal CA1 and DG regions in 6-month-old PS19 mice (Fig. 1S–T), indicating that abemaciclib treatment downregulates DYRK1A expression in PS19 mice. A future study will assess whether abemaciclib treatment modulates transcription levels (e.g., DYRK1A mRNA levels), protein degradation (e.g., ubiquitin–proteasome or lysosomal pathways), or kinase activity to reduce DYRK1A protein levels in PS19 mice. In addition, we did not directly assess pathological tau species; therefore, the relationship between the observed behavioral and neuroinflammatory changes and tau pathology should be interpreted with caution. We will investigate whether abemaciclib directly modulates pathological tau using tau-specific markers (e.g., AT8, Alz-50, or MC-1) in future studies.

Since abemaciclib administration significantly suppressed DYRK1A expression, we further examined the role of DYRK1A in the effects of abemaciclib administration on the cognitive function of human tau transgenic mice. Six-month-old PS19 mice were injected with AAV-CMV-Control shRNA or AAV-CMV-DYRK1A shRNA at bilateral hippocampal CA1. One week later, the mice were injected (i.p.) with vehicle (10% DMSO) or 30 mg/kg abemaciclib daily for 17 days. Western blot analysis showed that DYRK1A protein levels were lower in 6-month-old PS19 mice treated with control shRNA and abemaciclib than in PS19 mice treated with control shRNA and vehicle (Fig. 1U–V). However, DYRK1A protein levels were not further reduced in 6-month-old PS19 mice treated with DYRK1A shRNA and abemaciclib compared with PS19 mice treated with DYRK1A shRNA and vehicle (Fig. 1U-V). The results of Y-maze and NOR tests revealed that short-term spatial and recognition memory were enhanced in control shRNA and abemaciclib-treated 6-month-old PS19 mice compared to control shRNA and vehicle-treated PS19 mice (Fig. 1W-X). Moreover, DYRK1A shRNA and abemaciclib treatment improved recognition memory compared to control shRNA and vehicle treatment in 6-month-old PS19 mice (Fig. 1W–X). Importantly, abemaciclib treatment did not improve short-term and recognition memory in DYRK1A shRNA-treated 6-month-old PS19 mice (Fig. 1W-X).

The lack of additive effects and the absence of abemaciclib efficacy in PS19 mice in which DYRK1A was knocked down suggest that the positive effects of abemaciclib on short-term and recognition memory in 6-month-old human tau transgenic PS19 mice require DYRK1A. A potential alternative explanation for the lack of additive effects is pathway convergence: since both abemaciclib treatment and DYRK1A knockdown independently reduced DYRK1A levels and improved recognition memory, they may converge on a shared downstream pathway (Fig. 1U–V, X). However, this explanation does not fully account for the present findings, as the effect of abemaciclib treatment on cognitive function was abolished in DYRK1A-knockdown PS19 mice (Fig. 1X). Specifically, abemaciclib treatment did not produce any additional reduction in DYRK1A expression or improvement in cognitive function under DYRK1A-deficient conditions. If the two interventions were acting through parallel but converging pathways, some additive effect would be expected even in the absence of DYRK1A. Another possible explanation is a ceiling effect in which DYRK1A knockdown alone produces a maximal phenotype that masks any additional effect of abemaciclib treatment. However, the present findings do not support this possibility, as DYRK1A knockdown alone did not improve short-term spatial memory in the Y-maze test in 6-month-old PS19 mice (Fig. 1W). Collectively, our findings indicate that the lack of additive effects cannot be fully explained by pathway convergence or a ceiling effect. Rather, the absence of any detectable effect of abemaciclib treatment in DYRK1A-knockdown PS19 mice indicates that DYRK1A is required for cognitive improvement.

Abemaciclib directly inhibits DYRK1A with high potency (IC_50_ = 6 nM) comparable to that for its canonical targets CDK4 (IC_50_ = 2 nM) and CDK6 (IC_50_ = 10 nM) [6]. In the present study, we further showed that abemaciclib administration significantly decreased DYRK1A expression in the Entorhinal Cortex (EC) and hippocampus of 6-month-old PS19 mice and that abemaciclib-treated PS19 mice exhibited improved short-term and recognition memory in a DYRK1A-dependent manner (Fig. 1S–X). These findings support DYRK1A as the primary mediator of the cognitive effects of abemaciclib treatment in 6-month-old PS19 mice. Interestingly, a recent study reported that abemaciclib directly binds CaMKII and GSK3β and inhibits their kinase activities in a CDK4/6-independent manner, suggesting that CaMKII and GSK3β are direct targets of abemaciclib rather than downstream effectors [8]. In addition, DYRK1A, CaMKII, and GSK3β are known to regulate tau phosphorylation through parallel or partially overlapping mechanisms rather than a strictly hierarchical pathway. Specifically, DYRK1A phosphorylates tau at specific residues (e.g., Thr231), facilitating subsequent phosphorylation by GSK3β, whereas CaMKII phosphorylates tau at distinct residues (e.g., Ser262 and Ser356), indicating that it functions through an independent pathway [12, 13]. CDK4/6, CaMKII, and GSK3β may be considered secondary or parallel targets that contribute to broader kinase modulation. To further define the functional contributions of these secondary targets, future studies will use shRNA systems to investigate whether CDK4/6, CaMKII, and GSK3β are involved in regulating synaptic and cognitive functions in PS19 mice.

In summary, the present study demonstrates that the CDK4/CDK6 inhibitor abemaciclib downregulates microgliosis/astrogliosis in 6-month-old human tau transgenic PS19 mice. Importantly, abemaciclib administration improved short-term and recognition memory in a DYRK1A-dependent manner in 6-month-old PS19 mice. While abemaciclib has been reported to target multiple tau-related kinases, including CaMKII and GSK3β, the present results indicate that a distinct DYRK1A-dependent mechanism underlies its cognitive effects. Taken together, these findings provide insights into how multi-kinase targeting by abemaciclib may be leveraged for the treatment of neurodegenerative diseases.

## Supplementary Information

Supplementary Material 1.

## Data Availability

All data generated and/or analyzed during this study are included in this published article and its supplementary materials. Materials and methods are presented in the supplementary materials.

## Abbreviations

Aβ: Amyloid β
AD: Alzheimer’s disease
CDK-4/6: Cyclin-dependent kinase-4/6
DYRK1A: Dual specificity tyrosine-phosphorylation-regulated kinase 1A
NFT: Neurofibrillary tangle
