# Supplementary material for: The CDK4/6 inhibitor abemaciclib attenuates cognitive impairment and neuroinflammation via DYRK1A in human tau transgenic mice
Source: Mol Brain. 2026 Jun 1;19:60. doi: 10.1186/s13041-026-01318-x (PMC13439831; doi:10.1186/s13041-026-01318-x)
Supplement: Supplementary file 1 [file 13041_2026_1318_MOESM1_ESM.docx]

**The CDK4/6 inhibitor Abemaciclib attenuates cognitive impairment and neuroinflammation via DYRK1A in human tau transgenic mice**

**Hyun-ju Lee^1,2^ | Hyang-Sook Hoe^1,2,3,^***

^1^Department of Neural Development and Disease, Korea Brain Research Institute (KBRI), 61, Cheomdan-ro, Daegu, 41062, Republic of Korea; ^2^AI-based Neurodevelopmental Diseases Digital Therapeutics Group, Korea Brain Research Institute (KBRI), 61, Cheomdan-ro, Daegu, 41062, Republic of Korea; ^3^Department of Brain and Cognitive Sciences, Daegu Gyeongbuk Institute of Science & Technology, Daegu 42988, Republic of Korea

*Corresponding author

**Hyang-Sook Hoe, Ph.D**.: Department of Neural Development and Disease, Korea Brain Research Institute (KBRI), 61 Cheomdan-ro, Dong-gu, Daegu, Korea, 41068; E-mail: [sookhoe72@kbri.re.kr](mailto:sookhoe72@kbri.re.kr)

**Material and Methods**

Ethics Statement

All experiments were approved by the institutional biosafety committee (IBC) and performed in accordance with approved animal protocols of the Korea Brain Research Institute (KBRI, approval no. IACUC-19-00049, IACUC-22-00015).

**CDK4/CDK6 inhibitor abemaciclib mesylate**

The CDK4/CDK6 inhibitor abemaciclib mesylate was purchased from Selleck Chemicals (S7158, Houston, TX, USA) and MedKoo Biosciences, Inc. (Morrisville, NC, USA). The dose of abemaciclib mesylate was 30 mg/kg (i.p., dissolved in 10% DMSO) in *in vivo* experiments.

**Human mutant P301S tau transgenic (PS19) mice**

To examine whether abemaciclib modulates neuroinflammation and cognitive function, Human mutant P301S tau transgenic (PS19) mice were used (6 and 9 months old, B6;C3-Tg (Prnp-MAPT*P301S)PS19Vle/J; Stock No. 008169, Jackson Laboratory, Bar Harbor, ME, USA). The mice were maintained in a pathogen-free facility with a 12-h photoperiod in cages housing 3–4 mice each and had access to food and water *ad libitum*.

**Stereotaxic viral injection**

To investigate whether abemaciclib affects cognitive function through DYRK1A in aged PS19 mice, the mice were anesthetized [150 mg/kg 2,2,2-tribromoethanol, 2.5% v/v, i.p. (Sigma Aldrich, St. Louis, MO)] and AAV-CMV-Control shRNA-EGFP or AAV-CMV-mDYRK1A shRNA-EGFP (cat. no. shAAV-257590, Vector Biolabs, Malvern, PA, USA) was injected into the bilateral hippocampus at a 0° angle at the following stereotaxic coordinates from the bregma: -2.0 mm AP, ±1.5 mm ML, and -1.55 mm DV. The virus was bilaterally injected at a rate of 0.3 μL/min, and 1.0 μL was administered to each hemisphere using a 10-μL syringe (Hamilton, Reno, Nevada, USA). After surgery, sterile PBS (pH 7.4) was used to swab the wound, followed by suturing or clipping of the skin. The mice were allowed to recover for 3 weeks before further behavioral experiments.

**Behavioral tests**

To investigate whether abemaciclib alters learning and memory, 6- and 9-month-old PS19 mice were injected with abemaciclib (30 mg/kg, i.p.) or vehicle (10% DMSO) daily for 17 days and Y-maze and novel object recognition (NOR) tests were performed. In addition, to determine the effects of abemaciclib on learning and memory via DYRK1A, 6-month-old PS19 mice were injected with DYRK1A shRNA-virus or control shRNA virus at bilateral hippocampal CA1. One week after the injection, the mice were injected with vehicle (10% DMSO) or abemaciclib (30 mg/kg, i.p.) daily for 17 days and Y-maze and novel object recognition (NOR) tests were performed. Behavioral tests were conducted without blinding; however, data analyses were performed by an investigator blinded to the experimental groups

**Y-maze test**

The Y-maze test was used to evaluate hippocampal-dependent short-term spatial memory. The maze consisted of three arms (35 cm x 7 cm x 15 cm) joined in the center at angles of 120°. During the test, one mouse freely explored the arms for 5 min. A video camera was used to record spontaneous alternations, which were manually counted. Dividing the number of alternations by the number of alternation triads gave the alternation percentage.

**Novel object recognition (NOR) test**

To test the effects of abemaciclib on recognition memory, the NOR test was used. The NOR test comprised a training phase and a testing phase conducted in an open-field box (40 cm x 40 cm x 25 cm). In the training phase, two identical objects were placed in the box, and one mouse was placed in the box to freely explore it for 5 min. After each trial, odor cues were removed by thoroughly cleaning the box and objects with 70% ethanol. Twenty-four hours after the training phase, the testing phase was performed. The testing phase was identical to the training phase, except that one of the familiar objects was replaced with a novel object. The locations of the novel and familiar objects were counterbalanced throughout the trials. A video camera was used to record all trials. Pointing of the mouse's nose toward an object was considered exploration, and the exploration time for each object was counted manually to calculate the object preference (%): [Object preference (%) = Time _Novel_/(Time _Familiar_ + Time _Novel_) × 100].

**Golgi staining**

To examine whether abemaciclib affects dendritic spine formation, Golgi staining was performed after the behavioral tests with an FD Rapid GolgiStain Kit (FD Neurotechnologies, MD, USA) according to the manufacturer’s instructions. Then, the dendritic spine numbers in the apical oblique (AO) and basal shaft (BS) in the hippocampal CA1 were quantified using the software ImageJ (version 1.53e, National Institutes of Health, Bethesda, MD, USA).

**Immunofluorescence staining (IF)**

To investigate whether abemaciclib regulates neuroinflammatory responses and DYRK1A expression, 6-month-old PS19 mice were injected with abemaciclib (30 mg/kg, i.p.) or vehicle (10% DMSO) for 17 consecutive days and then anesthetized with 2,2,2-tribromoethanol (2.5% v/v, 150 mg/kg, i.p.; Sigma–Aldrich, St. Louis, MO, USA). The anesthetized PS19 mice were then subjected to transcardial perfusion with PBS followed by 4% paraformaldehyde (PFA) (Chembio, Seoul, Republic of Korea). The brains of the mice were removed and post-fixed at 4°C in 4% PFA overnight and then in 30% sucrose solution for 2 days. A cryostat (Leica CM1850, Wetzlar, Germany) was used to obtain coronal slices with a thickness of 35 µm, which were then permeabilized at room temperature for 1 h with PBS containing 0.2% Triton X-100 (PBST) and 10% normal goat serum. The brain sections from PS19 mice were immunostained for 24-72 h at 4°C with anti-Iba-1, anti-GFAP, or anti-DYRK1A antibodies diluted in PBST. After incubation with the primary antibody, the sections were washed with PBST three times. Incubation with the appropriate secondary antibody was then performed at room temperature for 2 h. Finally, the sections were washed three times with PBS and mounted in antifade mounting medium with DAPI (Vector Laboratories, Burlingame, CA, USA). Images of the sections were acquired by fluorescence microscopy (DMi8, Leica Microsystems, Wetzlar, Germany) and analyzed by the software ImageJ (version 1.53a, National Institutes of Health, Bethesda, MD, USA).

**Western blotting**

To test whether abemaciclib alters DYRK1A levels, 6-month-old PS19 mice were injected with DYRK1A or control shRNA virus at hippocampal CA1. One week after the injection, the mice were injected with vehicle (10% DMSO) or abemaciclib (30 mg/kg, i.p.) daily for 17 days. The hippocampus was then dissected, homogenized in RIPA lysis buffer (Merck Millipore, Billerica, MA, USA) containing 1% protease and phosphatase inhibitor cocktail (Thermo Scientific, Waltham, MA, USA), and incubated for 1 h on ice. The brain lysate was centrifuged at 20,000g for 15 min at 4 °C, and the supernatant was collected and stored at −80 °C until analysis. For western blot analysis, 10 μg of protein was heated for 5 min at 100°C, loaded onto a 8% SDS-polyacrylamide gel for separation by electrophoresis, and electrotransferred to a PVDF membrane (Millipore, Bedford, MA, USA). The membrane was blocked with 5% skim milk in TBST and then incubated overnight with the anti-DYRK1A antibody at 4°C. The membrane was then incubated with the horseradish peroxidase-conjugated secondary antibody for 1 h at RT. Finally, ECL solution (ATTO, Tokyo, Japan) was added for detection, and Fusion Capt Advance software (Vilber Lourmat, Eberhardzell, Germany) was used to acquire and analyze images.

**Statistical analysis**

GraphPad Prism 7 software (GraphPad Software, San Diego, CA, USA) was used for graph generation and statistical analysis. Data are presented as individual data points and the mean ± SEM. Student's *t* test was used for pairwise comparisons, and one-way analysis of variance (ANOVA) with Tukey’s or Newman-Keuls multiple-comparisons test was used for multiple comparisons. Asterisks indicate significance: * *p* < 0.05, ***p* < 0.01, and *** *p* < 0.001.

**Supplementary Table 1**. Statistical analysis results of the experiments in this study.

**Fig1A-B. Iba-1 intensity in the Entorhinal Cortex of 6-month-old PS19 mice**

| Table Analyzed | Data 1 |
| --- | --- |
|  |  |
| Column B | Data Set-B |
| vs. | vs. |
| Column A | Data Set-A |
|  |  |
| Unpaired t test |  |
| P value | 0.4747 |
| P value summary | ns |
| Significantly different (P < 0.05)? | No |
| One- or two-tailed P value? | Two-tailed |
| t, df | t=0.7224, df=36 |

**Fig1A-B. Iba-1 intensity in the Hippocampal CA1 region of 6-month-old PS19 mice**

| Table Analyzed | Data 1 |
| --- | --- |
|  |  |
| Column D | Data Set-D |
| vs. | vs. |
| Column C | Data Set-C |
|  |  |
| Unpaired t test |  |
| P value | 0.0241 |
| P value summary | * |
| Significantly different (P < 0.05)? | Yes |
| One- or two-tailed P value? | Two-tailed |
| t, df | t=2.355, df=36 |

**Fig1A-B. Iba-1 intensity in the Hippocampal DG region of 6-month-old PS19 mice**

| Table Analyzed | Data 1 |
| --- | --- |
|  |  |
| Column F | Data Set-F |
| vs. | vs. |
| Column E | Data Set-E |
|  |  |
| Unpaired t test |  |
| P value | 0.2363 |
| P value summary | ns |
| Significantly different (P < 0.05)? | No |
| One- or two-tailed P value? | Two-tailed |
| t, df | t=1.204, df=36 |

**Fig1A, C. Iba-1-labeled area in the Entorhinal Cortex of 6-month-old PS19 mice**

| Table Analyzed | Data 1 |
| --- | --- |
|  |  |
| Column B | Data Set-B |
| vs. | vs. |
| Column A | Data Set-A |
|  |  |
| Unpaired t test |  |
| P value | 0.0103 |
| P value summary | * |
| Significantly different (P < 0.05)? | Yes |
| One- or two-tailed P value? | Two-tailed |
| t, df | t=2.708, df=36 |

**Fig1A, C. Iba-1-labeled area in the Hippocampal CA1 region of 6-month-old PS19 mice**

| Table Analyzed | Data 1 |
| --- | --- |
|  |  |
| Column D | Data Set-D |
| vs. | vs. |
| Column C | Data Set-C |
|  |  |
| Unpaired t test |  |
| P value | 0.0582 |
| P value summary | ns |
| Significantly different (P < 0.05)? | No |
| One- or two-tailed P value? | Two-tailed |
| t, df | t=1.956, df=36 |

**Fig1A, C. Iba-1-labeled area in the Hippocampal DG region of 6-month-old PS19 mice**

| Table Analyzed | Data 1 |
| --- | --- |
|  |  |
| Column F | Data Set-F |
| vs. | vs. |
| Column E | Data Set-E |
|  |  |
| Unpaired t test |  |
| P value | 0.2657 |
| P value summary | ns |
| Significantly different (P < 0.05)? | No |
| One- or two-tailed P value? | Two-tailed |
| t, df | t=1.131, df=36 |

**Fig1D-E. GFAP intensity in the Entorhinal Cortex of 6-month-old PS19 mice**

| Table Analyzed | GFAP |
| --- | --- |
|  |  |
| Column B | Data Set-B |
| vs. | vs. |
| Column A | Data Set-A |
|  |  |
| Unpaired t test |  |
| P value | 0.0047 |
| P value summary | ** |
| Significantly different (P < 0.05)? | Yes |
| One- or two-tailed P value? | Two-tailed |
| t, df | t=3.023, df=35 |

**Fig1D-E. GFAP intensity in the Hippocampal CA1 region**

| Table Analyzed | GFAP |
| --- | --- |
|  |  |
| Column D | Data Set-D |
| vs. | vs. |
| Column C | Data Set-C |
|  |  |
| Unpaired t test |  |
| P value | 0.0598 |
| P value summary | ns |
| Significantly different (P < 0.05)? | No |
| One- or two-tailed P value? | Two-tailed |
| t, df | t=1.949, df=33 |
|  |  |

**Fig1D-E. GFAP intensity in the Hippocampal DG region of 6-month-old PS19 mice**

| Table Analyzed | GFAP |
| --- | --- |
|  |  |
| Column F | Data Set-F |
| vs. | vs. |
| Column E | Data Set-E |
|  |  |
| Unpaired t test |  |
| P value | 0.3900 |
| P value summary | ns |
| Significantly different (P < 0.05)? | No |
| One- or two-tailed P value? | Two-tailed |
| t, df | t=0.8711, df=33 |

**Fig1D, F. GFAP-labeled area in the Entorhinal Cortex of 6-month-old PS19 mice**

| Table Analyzed | GFAP |
| --- | --- |
|  |  |
| Column B | Data Set-B |
| vs. | vs. |
| Column A | Data Set-A |
|  |  |
| Unpaired t test |  |
| P value | 0.0121 |
| P value summary | * |
| Significantly different (P < 0.05)? | Yes |
| One- or two-tailed P value? | Two-tailed |
| t, df | t=2.647, df=35 |

**Fig1D, F. GFAP-labeled area in the Hippocampal CA1 region of 6-month-old PS19 mice**

| Table Analyzed | GFAP |
| --- | --- |
|  |  |
| Column D | Data Set-D |
| vs. | vs. |
| Column C | Data Set-C |
|  |  |
| Unpaired t test |  |
| P value | 0.0884 |
| P value summary | ns |
| Significantly different (P < 0.05)? | No |
| One- or two-tailed P value? | Two-tailed |
| t, df | t=1.756, df=33 |

**Fig1D, F. GFAP-labeled area in the Hippocampal DG region of 6-month-old PS19 mice**

| Table Analyzed | GFAP |
| --- | --- |
|  |  |
| Column F | Data Set-F |
| vs. | vs. |
| Column E | Data Set-E |
|  |  |
| Unpaired t test |  |
| P value | 0.5367 |
| P value summary | ns |
| Significantly different (P < 0.05)? | No |
| One- or two-tailed P value? | Two-tailed |
| t, df | t=0.6243, df=33 |

**Fig1G-H. Y maze Alternation in 6-month-old PS19 mice**

| Table Analyzed | Y-maze |
| --- | --- |
|  |  |
| Column B | Data Set-B |
| vs. | vs. |
| Column A | Data Set-A |
|  |  |
| Unpaired t test |  |
| P value | 0.0012 |
| P value summary | ** |
| Significantly different (P < 0.05)? | Yes |
| One- or two-tailed P value? | Two-tailed |
| t, df | t=3.995, df=15 |

**Fig1G-H. Y maze Total arm entry in 6-month-old PS19 mice**

| Table Analyzed | Y-maze |
| --- | --- |
|  |  |
| Column D | Data Set-D |
| vs. | vs. |
| Column C | Data Set-C |
|  |  |
| Unpaired t test |  |
| P value | 0.0025 |
| P value summary | ** |
| Significantly different (P < 0.05)? | Yes |
| One- or two-tailed P value? | Two-tailed |
| t, df | t=3.632, df=15 |

**Fig1G, I. Y maze Alternation in 9-month-old PS19 mice**

| Table Analyzed | Y-maze |
| --- | --- |
|  |  |
| Column B | Data Set-B |
| vs. | vs. |
| Column A | Data Set-A |
|  |  |
| Unpaired t test |  |
| P value | 0.0417 |
| P value summary | * |
| Significantly different (P < 0.05)? | Yes |
| One- or two-tailed P value? | Two-tailed |
| t, df | t=2.176, df=20 |

**Fig1G, I. Y maze Total arm entry in 9-month-old PS19 mice**

| Table Analyzed | Y-maze |
| --- | --- |
|  |  |
| Column D | Data Set-D |
| vs. | vs. |
| Column C | Data Set-C |
|  |  |
| Unpaired t test |  |
| P value | 0.4961 |
| P value summary | ns |
| Significantly different (P < 0.05)? | No |
| One- or two-tailed P value? | Two-tailed |
| t, df | t=0.6933, df=20 |

**Fig1J, K. Object Preference of NOR Training in 6-month-old PS19 mice**

| Table Analyzed | NORT |
| --- | --- |
|  |  |
| Column B | Data Set-B |
| vs. | vs. |
| Column A | Data Set-A |
|  |  |
| Unpaired t test |  |
| P value | 0.4414 |
| P value summary | ns |
| Significantly different (P < 0.05)? | No |
| One- or two-tailed P value? | Two-tailed |
| t, df | t=0.7922, df=14 |

**Fig1J, K. Object Preference of NOR Test in 6-month-old PS19 mice**

| Table Analyzed | NOR |
| --- | --- |
|  |  |
| Column D | Data Set-D |
| vs. | vs. |
| Column C | Data Set-C |
|  |  |
| Unpaired t test |  |
| P value | 0.0322 |
| P value summary | * |
| Significantly different (P < 0.05)? | Yes |
| One- or two-tailed P value? | Two-tailed |
| t, df | t=2.378, df=14 |

**Fig1J, L. Object Preference of NOR Training in 9-month-old PS19 mice**

| Table Analyzed | NORT |
| --- | --- |
|  |  |
| Column B | Data Set-B |
| vs. | vs. |
| Column A | Data Set-A |
|  |  |
| Unpaired t test |  |
| P value | 0.5040 |
| P value summary | ns |
| Significantly different (P < 0.05)? | No |
| One- or two-tailed P value? | Two-tailed |
| t, df | t=0.6811, df=19 |

**Fig1J, L. Object Preference of NOR Test in 9-month-old PS19 mice**

| Table Analyzed | NORT |
| --- | --- |
|  |  |
| Column D | Data Set-D |
| vs. | vs. |
| Column C | Data Set-C |
|  |  |
| Unpaired t test |  |
| P value | 0.0025 |
| P value summary | ** |
| Significantly different (P < 0.05)? | Yes |
| One- or two-tailed P value? | Two-tailed |
| t, df | t=3.487, df=19 |

**Fig1M-O. Dendritic spine numbers in the Hippocampal CA1 region (AO) of 6-month-old PS19 mice**

| Table Analyzed | AO |
| --- | --- |
|  |  |
| Column B | Abe |
| vs. | vs. |
| Column A | **Veh** |
|  |  |
| Unpaired t test |  |
| P value | <0.0001 |
| P value summary | **** |
| Significantly different (P < 0.05)? | Yes |
| One- or two-tailed P value? | Two-tailed |
| t, df | t=5.506, df=38 |

**Fig1N-O. Dendritic spine numbers in the Hippocampal CA1 region (BS) of 6-month-old PS19 mice**

| Table Analyzed | BS |
| --- | --- |
|  |  |
| Column B | Abe |
| vs. | vs. |
| Column A | **Veh** |
|  |  |
| Unpaired t test |  |
| P value | 0.0283 |
| P value summary | * |
| Significantly different (P < 0.05)? | Yes |
| One- or two-tailed P value? | Two-tailed |
| t, df | t=2.280, df=38 |

**Fig1P-R. Dendritic spine numbers in the Hippocampal CA1 region (AO) of 9-month-old PS19 mice**

| Table Analyzed | Data 1 |
| --- | --- |
|  |  |
| Column B | S7158 |
| vs. | vs. |
| Column A | Cont |
|  |  |
| Unpaired t test |  |
| P value | 0.0011 |
| P value summary | ** |
| Significantly different (P < 0.05)? | Yes |
| One- or two-tailed P value? | Two-tailed |
| t, df | t=3.539, df=38 |

**Fig1P-R. Dendritic spine numbers in the Hippocampal CA1 region (BS) of 9-month-old PS19 mice**

| Table Analyzed | Data 1 |
| --- | --- |
|  |  |
| Column B | S7158 |
| vs. | vs. |
| Column A | Cont |
|  |  |
| Unpaired t test |  |
| P value | <0.0001 |
| P value summary | **** |
| Significantly different (P < 0.05)? | Yes |
| One- or two-tailed P value? | Two-tailed |
| t, df | t=5.019, df=38 |

**Fig1S-T. DYRK1A fluorescence intensity in the Entorhinal Cortex of 6-month-old PS19 mice**

| Table Analyzed | dyrk1a-EC |
| --- | --- |
|  |  |
| Column B | + - |
| vs. | vs. |
| Column A | - - |
|  |  |
| Unpaired t test |  |
| P value | <0.0001 |
| P value summary | **** |
| Significantly different (P < 0.05)? | Yes |
| One- or two-tailed P value? | Two-tailed |
| t, df | t=6.094, df=38 |

**Fig1S-T. DYRK1A fluorescence intensity in the Hippocampal CA1 region of 6-month-old PS19 mice**

| Table Analyzed | dyrk1a-ca1 |
| --- | --- |
|  |  |
| Column B | + - |
| vs. | vs. |
| Column A | - - |
|  |  |
| Unpaired t test |  |
| P value | <0.0001 |
| P value summary | **** |
| Significantly different (P < 0.05)? | Yes |
| One- or two-tailed P value? | Two-tailed |
| t, df | t=4.934, df=38 |

**Fig1S-T. DYRK1A fluorescence intensity in the Hippocampal DG region of 6-month-old PS19 mice**

| Table Analyzed | dyrk1a- DG |
| --- | --- |
|  |  |
| Column B | + - |
| vs. | vs. |
| Column A | - - |
|  |  |
| Unpaired t test |  |
| P value | <0.0001 |
| P value summary | **** |
| Significantly different (P < 0.05)? | Yes |
| One- or two-tailed P value? | Two-tailed |
| t, df | t=4.524, df=38 |

**Fig1U-V. DYRK1A levels in the Hippocampus of 6-month-old PS19 mice**

| Number of families | 1 |  |  |  |  |
| --- | --- | --- | --- | --- | --- |
| Number of comparisons per family | 6 |  |  |  |  |
| Alpha | 0.05 |  |  |  |  |
|  |  |  |  |  |  |
| Newman-Keuls multiple comparisons test | Mean Diff. | Below threshold? | Summary |  |  |
| Column A vs. Column B | 36.29 | Yes | * |  | A-B |
| Column A vs. Column C | 58.67 | Yes | ** |  | A-C |
| Column A vs. Column D | 59.30 | Yes | ** |  | A-D |
| Column B vs. Column C | 22.38 | No | ns |  | B-C |
| Column B vs. Column D | 23.01 | No | ns |  | B-D |
| Column C vs. Column D | 0.6349 | No | ns |  | C-D |

**Fig1W. Y maze Alternation in 6-month-old PS19 mice**

| Table Analyzed | Y maze |
| --- | --- |
|  |  |
| Column B | Data Set-B |
| vs. | vs. |
| Column A | Data Set-A |
|  |  |
| Unpaired t test |  |
| P value | 0.0397 |
| P value summary | * |
| Significantly different (P < 0.05)? | Yes |
| One- or two-tailed P value? | Two-tailed |
| t, df | t=2.286, df=13 |
| Table Analyzed | Y maze |
|  |  |
| Column D | Data Set-D |
| vs. | vs. |
| Column C | Data Set-C |
|  |  |
| Unpaired t test |  |
| P value | 0.7828 |
| P value summary | ns |
| Significantly different (P < 0.05)? | No |
| One- or two-tailed P value? | Two-tailed |
| t, df | t=0.2815, df=13 |
|  |  |

**Fig1X. Object preference of NOR test in 6-month-old PS19 mice**

| Number of families | 1 |  |  |  |  |  |
| --- | --- | --- | --- | --- | --- | --- |
| Number of comparisons per family | 6 |  |  |  |  |  |
|  |  |  |  |  |  |  |
| Alpha | 0.05 |  |  |  |  |  |
|  |  |  |  |  |  |  |
| Tukey's multiple comparisons test | Mean Diff. | 95.00% CI of diff. | Below threshold? | Summary | Adjusted P Value |  |
| Column A vs. Column B | -16.83 | -32.53 to -1.135 | Yes | * | 0.0322 | A-B |
| Column A vs. Column C | -17.72 | -32.88 to -2.550 | Yes | * | 0.0176 | A-C |
| Column A vs. Column D | -20.05 | -35.75 to -4.354 | Yes | ** | 0.0086 | A-D |
| Column B vs. Column C | -0.8824 | -16.58 to 14.82 | No | ns | 0.9987 | B-C |
| Column B vs. Column D | -3.219 | -19.43 to 12.99 | No | ns | 0.9472 | B-D |
| Column C vs. Column D | -2.336 | -18.03 to 13.36 | No | ns | 0.9765 | C-D |
|  |  |  |  |  |  |  |
